# 

**Published:** 1888-06

**Authors:** 


### *PUBLICATIONS RECEIVED.*

From G. P. Engelhard & Co., Chicago:
*The Medical Standard.* March—May, 1888.

From The Medical Society of the County of Kings:
*The Brooklyn Medical Journal.* February, 1888.

From John Carnrick, New York:
*The Dietetic Gazette.* April, 1888.

From P. Blakiston, Son & Co., Philadelphia:
*The Polyclinic.* March—May, 1888.

From John Bale & Sons, London:
*The Principles of Cancer and Tumour Formation.* By W. Roger Williams. 1888.

From J. & A. Churchill, London:
*The Waters of Plombières* (Vosges). By Dr. Bottentuit. 1888.
*The Milroy Lectures.* By Robert Lawson, LL.D. 1888.
*The Localisation of the Lesions of Phthisis in Relation to Diagnosis and Prognosis.* By J. Kingston Fowler, M.D. 1888.
*The Electric Illumination of the Bladder and Urethra.* By E. Hurry Fenwick. 1888.
*Deviations of the Nasal Septum.* By George Stoker. 1888.

From H. K. Lewis, London:
*Inebriety.* By Norman Kerr, M.D. 1888.
*Gonorrhœal Infection in Women.* By William Japp Sinclair, M.A., M.D. 1888.
*Cancer of the Uterus.* By John Williams, M.D. 1888.
*Anæsthetics.* By Dudley Wilmot Buxton, M.D., B.S. 1888.
*Diseases of Women.* By Arthur H. N. Lewers, M.D. 1888.
*Handbook of Diseases of the Eye.* By Henry R. Swanzy, A.M., M.B. Second Edition. 1888.
*Introduction to the British Pharmacopœia.* By Rawdon Macnamara. 1888.
*The Sectional Anatomy of Congenital Cœcal Hernia.* By E. H. Bennett, M.D., and D. J. Cunningham, M.D. 1888.

From Sampson Low & Co., London:
*The Nursing Record.* Vol. i., No. 2. April 12th, 1888.

From Young J. Pentland, Edinburgh:
*Intracranial Tumours.* By Byrom Bramwell, M.D. 1888.

FOUNDED 1831.

### H. W. CARTER & CO.'S
### HIGH CLASS MINERAL WATERS.

JOHN ATTFIELD, F.C.S., London; A. HILL, M.D., Birmingham; W.W. STODDART, F.C.S., Bristol; F. F. PERKINS, F.C.S., Exeter; GRANVILLE SHARPE, F.C.S., London; E. W. T. JONES, F.C.S., Wolverhampton; and other Experts, have testified to the purity of these celebrated Aerated Waters.

*The Original West of England Manufactory:*
OLD REFINERY, ST. PAUL'S, BRISTOL.

### Bristol Medico-Chirurgical Journal.

### JUNE, 1888.

*Cases for binding, price 7d. each, may be had of the Publisher,*
J. W. ARROWSMITH, *Quay Street, Bristol.*

### Bristol Medico-Chirurgical Society.

---

### President.

L. M. GRIFFITHS.

### Honorary Secretary and President-Elect.

R. SHINGLETON SMITH, M.D., B.Sc. Lond.

### Committee.

*Past Presidents.*
R. W. COE.
AUGUSTIN PRICHARD, M.D. Berlin.
J. G. SWAYNE, M.D. Lond.
E. LONG FOX, M.D. Oxon.
JAMES G. DAVEY, M.D. St. And.
W. JOHNSTONE FYFFE, M.D. Dub.
G. F. BURDER, M.D. Aberd.
W. H. SPENCER, M.D., M.A. Cantab.
F. POOLE LANSDOWN.

F. RICHARDSON CROSS, M.B. Lond.
NELSON C. DOBSON.
A. J. HARRISON, M.B. Lond.
W. H. HARSANT.
E. MARKHAM SKERRITT, M.D., B.S., B.A. Lond.
J. GREIG SMITH, M.B., C.M., M.A. Aberd., F.R.S.E.

Medical Men wishing to join the Society are requested to communicate with the Honorary Secretary, Dr. R. SHINGLETON SMITH, 9 Richmond Hill, Clifton, Bristol. The Annual Subscription is 10/6.

---

*Extracts from Journal By-laws.*

4.—The *Journal* shall be delivered free to Subscribers and also to Members of the Society whose subscriptions are not in arrear.

6.—The Annual Subscription to the *Journal* for those who pay before the 1st of July shall be 5/-, post free, or 6/- if paid after that date.

---

Communications intended for insertion in the *Journal* should be sent to the Editor, J. GREIG SMITH, M.A., F.R.S.E., 16 Victoria Square, Clifton, Bristol.

Books for Review, Exchange Journals, and communications referring to the delivery of the *Journal* should be sent to the Assistant-Editor, L. M. GRIFFITHS, 9 Gordon Road, Clifton, Bristol, to whom Subscriptions are to be paid *in advance*.

Authors requiring Reprints of their Papers should communicate with the Publisher.

Cases for Binding Vols. I., II., III., IV., and V. are now ready, and may be obtained, price 7*d.* each (postage 2*d.* extra), upon application to J. W. ARROWSMITH, Quay Street, Bristol; or the *Journal* will be bound, including Case, for 1/3 per volume.

